# Global Gene Expression Shift during the Transition from Early Neural Development to Late Neuronal Differentiation in *Drosophila melanogaster*

**DOI:** 10.1371/journal.pone.0097703

**Published:** 2014-05-15

**Authors:** Rafael Cantera, María José Ferreiro, Ana María Aransay, Rosa Barrio

**Affiliations:** 1 Zoology Department, Stockholm University, Stockholm, Sweden; 2 Developmental Neurobiology, IIBCE, Montevideo, Uruguay; 3 Genome Analysis Platform, CIC bioGUNE and CIBERehd, Derio, Spain; 4 Functional Genomics, CIC bioGUNE, Derio, Spain; Instituto de Neurociencias, CSIC and UMH, Spain

## Abstract

Regulation of transcription is one of the mechanisms involved in animal development, directing changes in patterning and cell fate specification. Large temporal data series, based on microarrays across the life cycle of the fly *Drosophila melanogaster*, revealed the existence of groups of genes which expression increases or decreases temporally correlated during the life cycle. These groups of genes are enriched in different biological functions. Here, instead of searching for temporal coincidence in gene expression using the entire genome expression data, we searched for temporal coincidence in gene expression only within predefined catalogues of functionally related genes and investigated whether a catalogue's expression profile can be used to generate larger catalogues, enriched in genes necessary for the same function. We analyzed the expression profiles from genes already associated with early neurodevelopment and late neurodifferentiation, at embryonic stages 16 and 17 of *Drosophila* life cycle. We hypothesized that during this interval we would find global downregulation of genes important for early neuronal development together with global upregulation of genes necessary for the final differentiation of neurons. Our results were consistent with this hypothesis. We then investigated if the expression profile of gene catalogues representing particular processes of neural development matched the temporal sequence along which these processes occur. The profiles of genes involved in patterning, neurogenesis, axogenesis or synaptic transmission matched the prediction, with largest transcript values at the time when the corresponding biological process takes place in the embryo. Furthermore, we obtained catalogues enriched in genes involved in temporally matching functions by performing a genome-wide systematic search for genes with their highest expression levels at the corresponding embryonic intervals. These findings imply the use of gene expression data in combination with known biological information to predict the involvement of functionally uncharacterized genes in particular biological events.

## Introduction

Regulation of transcription is the first step in the regulation of protein expression, which determines cell fate and patterning during development, working under the control of several signaling pathways. However, the variety and complexity of post-transcriptional, translational and post-translational modifications indicate that gene expression profiles will often have very uncertain predictive value for the biological consequences that must follow the change in transcription. The massive increment in expression data, including relatively dense temporal data series across the life cycle of the fruit fly *Drosophila melanogaster* and other organisms enables us to address this question.

Clusters of genes that increase or decrease their expression within well-defined temporal windows along the life cycle of *Drosophila melanogaster* have been identified using DNA microarrays [1]–[4]. The finest temporal resolution for this type of analysis is presently available for the embryonic phase of development, where RNA samples were taken at intervals of one or two hours and monitored using microarrays representing either 30% of the genome [1], [4] or the entire genome [2], as well as using deep-sequencing [5].

Three basic classes of genes were defined according to their coherent pattern of temporal expression: downregulated, upregulated or transiently expressed [2], [3]. When the composition of gene clusters showing coordinated changes in expression was analyzed with regards to tissue expression and functional annotation, it became clear that changes in transcription levels were, in most cases, roughly co-temporal with global and broad developmental processes such as the specification of the body plan, the germ band elongation, the dorsal closure or the germ band retraction [2], [3]. A few groups were enriched with functional categories corresponding to processes with high spatial and temporal restrictions. For instance, a group enriched in genes important for muscle formation was found to be upregulated at the end of embryonic and pupal stages, when larval and adult muscles are formed, respectively [1]. Conversely, a group of muscle genes was found to be downregulated at early steps during metamorphosis, when most larval muscles are degraded [4]. Additionally, groups enriched in genes associated with cuticle secretion showed transient, short and substantial transcriptional upregulation during late embryonic development, at the time when the cuticle is secreted by the epidermis [2], [3].

These and other examples demonstrate that useful information for developmental studies of single tissues or organs can be obtained from temporal data series based in RNA extracted from whole organisms. The nervous system is a good candidate organ to test this approach because it is relatively large in the embryonic context, it expresses a great proportion of the genome and it is one of the best studied tissues during embryonic development [6], [7].

Detailed and extensive information is available for the molecular and cellular mechanisms that specify the neurogenic zone, the generation and specification of stem cells, the control of proliferation and programmed cell death, the generation of cell diversity and the differentiation of glial cells and neurons. These processes culminate with the establishment of functional neuronal networks used by the larva to hatch from the egg shell and start living as a free-moving animal, and can be subdivided into minor components that develop either in a temporal sequence or in parallel. For example, neuronal differentiation requires previous specification of undifferentiated cells to become neurons or glia [8], followed by axonal growth [9], [10], dendritic growth and branching [11], [12] and, finally, by the initiation of synaptic activity [13]. Each step in this sequence is controlled, at least in part, by specific pathways and combinations of several proteins, acting either once or at multiple times. For instance, the *dpp* (TGF-β/BMP) pathway contributes to the control of several developmental processes separated in space and time: the determination of the ventral neurogenic zone that produces the nerve cord in the ventral side of the embryo; the development of peripheral neurons in the lateral and dorsal region [14]; the growth and maintenance of the neuromuscular junction during larval life [15], [16] and the development of the eye under embryonic, larval and pupal life [17]–[19].

Gene clusters with coherent temporal changes in expression have not been extensively studied under the context of nervous system development. Two clusters significantly enriched in genes annotated with terms related to nervous system were previously reported. A cluster of 153 genes (Group II:a in Figure 2 in [2]) showed a sharp increase in transcription at the time of gastrulation (just before the first neural stem cells are formed) and was downregulated at 12 hours after egg laying (AEL). The second cluster composed of 304 genes had a peak at 6.2 hours of development AEL and extended for 7.1 hours (Cluster #7, Figure 3 in [3]). In both cases, the duration of the peaks spans over several distinct developmental steps. Therefore, these data lack enough temporal resolution and the only conclusion that can be made is that there is a good temporal correlation between early phases of neural development and an increased transcription of several genes, some of which are already defined as important for the nervous system.

Here, using mRNA-Seq data obtained from whole *Drosophila melanogaster* wild type embryos, we analyzed the expression of several gene catalogues generated to represent biological processes occurring along a relatively well-defined temporal sequence during neural development. First we questioned two major catalogues, representing either the early phases of neurodevelopment (which for the sake of simplicity will be referred to as “Neurodevelopment”) or a late phase of neurodevelopment, corresponding to late neurodifferentiation (hereafter called “Neurodifferentiation”). We found that the expression profile of each catalogue mirrored the dynamics of the corresponding biological process along an almost perfect temporal sequence. We also found that within each catalogue, many of the genes changed transcript levels simultaneously in a way that is coherent with the biological functions controlled by these genes. Furthermore, by genome-wide systematic search for genes with a particular gene expression profile, we obtained catalogues enriched in genes involved in the biological functions corresponding to the temporal frame chosen, demonstrating the predictive capacity of our analysis. The same approach can be applied to other tissues or developmental processes.

## Results

### A global switch in gene expression correlates with the transition from early neurodevelopment to late neurodifferentiation

**Figure 1:**
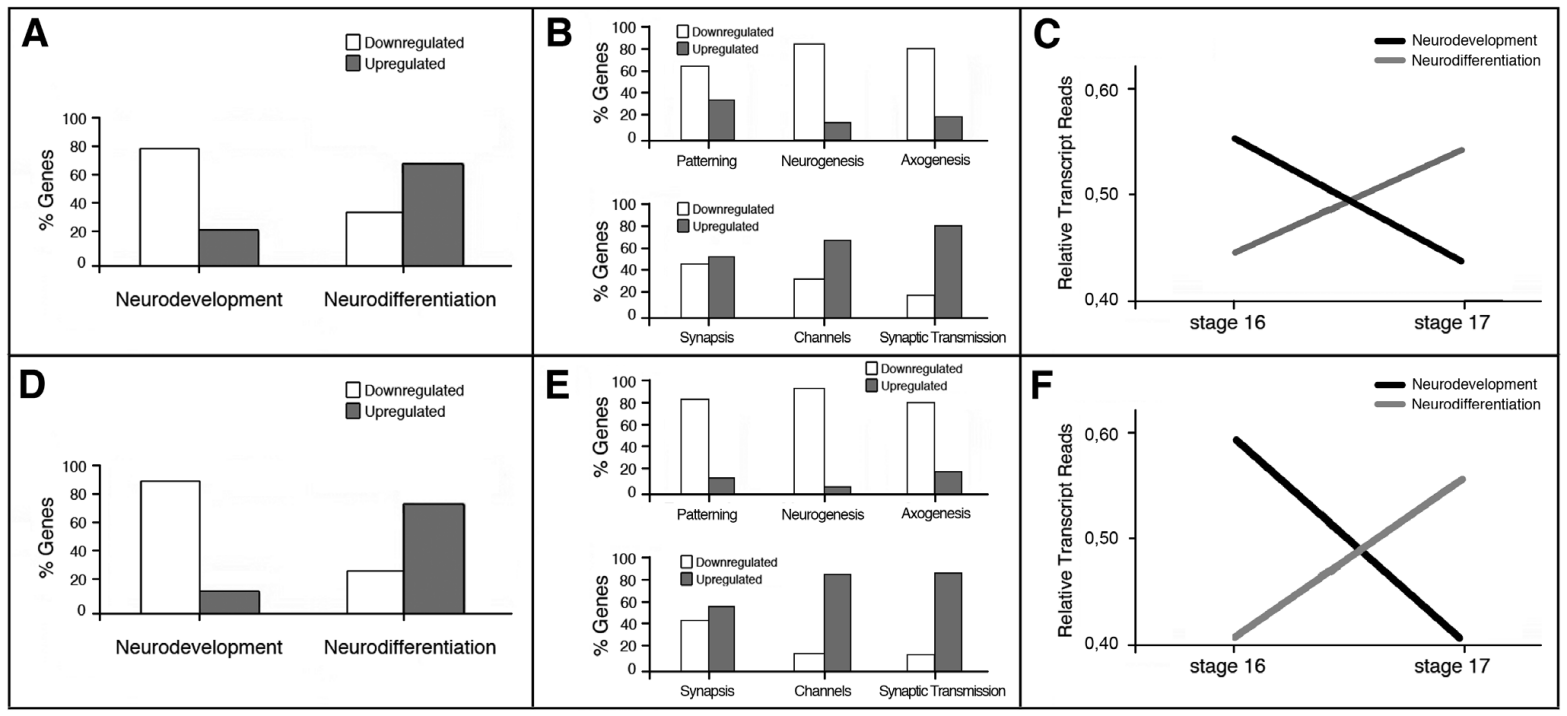
The transition from embryonic stage 16 to 17 in *Drosophila* correlates with downregulation of genes involved in nervous system early development and upregulation of genes involved in late neuronal differentiation. **A, B, D, E**: Graphical representation of the percentage of genes from Neurodevelopment and Neurodifferentiation catalogues that are up or downregulated at the transition from embryonic stage 16 to 17. C, **F**: Relative number of total transcript reads of the genes belonging to the Neurodevelopment or Neurodifferentiation catalogues plotted *versus* developmental time in a 0 to 1 scale. Transcriptome data from Ferreiro et al. [23] (**A–C**) or from Graveley et al. [5] (**D–F**) were used for the analysis.

The first synaptic activity in *Drosophila melanogaster* has been registered in embryonic motor neurons at approximately 14 hours post-fertilization [20] and at about 16 hours in interneurons [21]. Since synaptic activity matures rapidly during the following 2–3 hours [20]–[22], and the larvae hatches a few hours later with a functional nervous system, thousands of synapses must be rapidly formed during this period. This suggests that the early phases of neural development culminate around 14–16 hours of embryonic development, a time where the final steps of neuronal differentiation will intensify. A comparison of the *Drosophila melanogaster* wild type embryonic transcriptome at early stage 16 (13–14 hours AEL) and early stage 17 (17–18 hours AEL) showed that during this interval 1687 genes are upregulated and 312 are downregulated [23]. To test the hypothesis that the transition from embryonic stage 16 to 17 comprises global downregulation of genes important for early neuronal development and coherent upregulation of genes necessary for the final differentiation of neurons, we first generated two gene catalogues representing either the early stages of development (Neurodevelopment) or late stages of differentiation (Neurodifferentiation) (Table S1; see Material and Methods for a detailed explanation of the catalogues). The gene coverage of these catalogues did not pretend to be exhaustive, but each contained 200 of the genes known to be important for these functions. Moreover, each catalogue comprised sub-catalogues of genes important for a variety of more particular aspects of either early neurodevelopment or late neurodifferentiation. The analysis of the data previously generated [23] revealed that 79% of the early neurodevelopmental genes have lower transcription levels at stage 17 compared with stage 16 (Figure 1A). Conversely, 67% of the genes of the Neurodifferentiation catalogue show higher transcription levels at stage 17 (Figure 1A). The same general trend was obtained when the Neurodevelopment catalogue was subdivided into three sub-catalogues representing Patterning, Neurogenesis and Axogenesis phases (see below for explanation of the sub-catalogues) and when the Neurodifferentiation catalogue was subdivided into three sub-catalogues representing Synapses, Ion Channels and Synaptic Transmission (Figure 1B). The total number of transcripts' reads confirms this general trend, as it shows a global downregulation for Neurodevelopment genes together with an upregulation of Neurodifferentiation genes in the transition from early embryonic stage 16 to 17 (Figure 1C).

We applied the same method to an independent temporal data series, produced by the modENCODE project, which generated mRNA-Seq expression data from *Drosophila melanogaster* RNA samples collected throughout embryonic development at 2-hours intervals [5]. As a substitute for the two samples tested above, we selected the samples labeled “12–14 hours” and “18–20 hours” [5], because they represent the best-fitting time points for the “early embryonic stage 16” and “early embryonic stage 17” samples of Ferreiro et al. [23]. Though the methods, the genotypes of the samples and the time points do not match perfectly, we found good overall coincidence relative to the percentage of genes upregulated or downregulated at the transition from stage 16 to 17 (Figure 1D, E), as well as to the variation of total transcripts' reads (Figure 1F). During the transition from 12–14 hours to 18–20 hours 88% of the genes from the Neurodevelopment catalogue are downregulated and 74% of the genes from Neurodifferentiation catalogue are upregulated. These data reinforce the idea that the transition from stage 16 to 17 implies the downregulation of genes involved in early neural development and the upregulation of genes involved in late neuronal differentiation.

**Figure 2:**
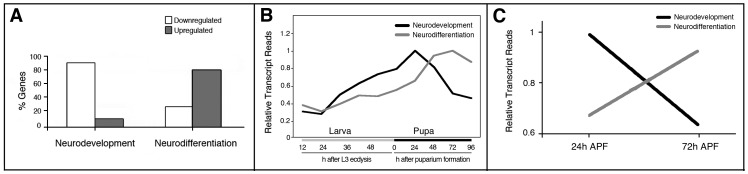
Transcriptional shift of genes involved in either early neurodevelopment or late neurodifferentiation during *Drosophila* metamorphosis. **A–C**: Graphical representation of the percentage of genes from the Neurodevelopment and Neurodifferentiation catalogues that are up or downregulated at the transition from larval to pupal stages. Transcriptome data are from Graveley et al. [5] **A**: Percentage of genes that are either up or downregulated between the first and third day of pupal development. **B**: The expression profile of Neurodevelopment and Neurodifferentiation catalogues along 12-hours intervals across the last phase of larval life (third instar) and every 24 hours across pupal life. **C**: Comparison of total transcripts of the Neurodevelopment or Neurodifferentiation catalogues shown as relative number of total reads plotted *versus* developmental time in a 0 to 1 scale.

During larval life, neural stem cells resume their mitotic activity [24] and produce a large progeny of cells that remains in a non-differentiated state until the beginning of metamorphosis, when they coordinately start their adult differentiation [25]. We then tested the hypothesis that a second shift from neurodevelopment to neurodifferentiation may occur during metamorphosis. We used the large temporal series of modENCODE [5] to compare the expression profiles of the Neurodevelopment and Neurodifferentiation catalogues during the last larval stage (third instar) and pupal stages. During pupal development it appears to be a recapitulation of the embryonic shift described above, with changes of similar magnitude in the percentage of genes up- or downregulated (Figure 2A). We found an increase in the total number of transcripts during early metamorphosis for both catalogues and observed that Neurodevelopment transcripts start to decline when those of Neurodifferentiation increase (Figure 2B, C).

### Temporal correlation of expression in functional gene groups

**Figure 3:**
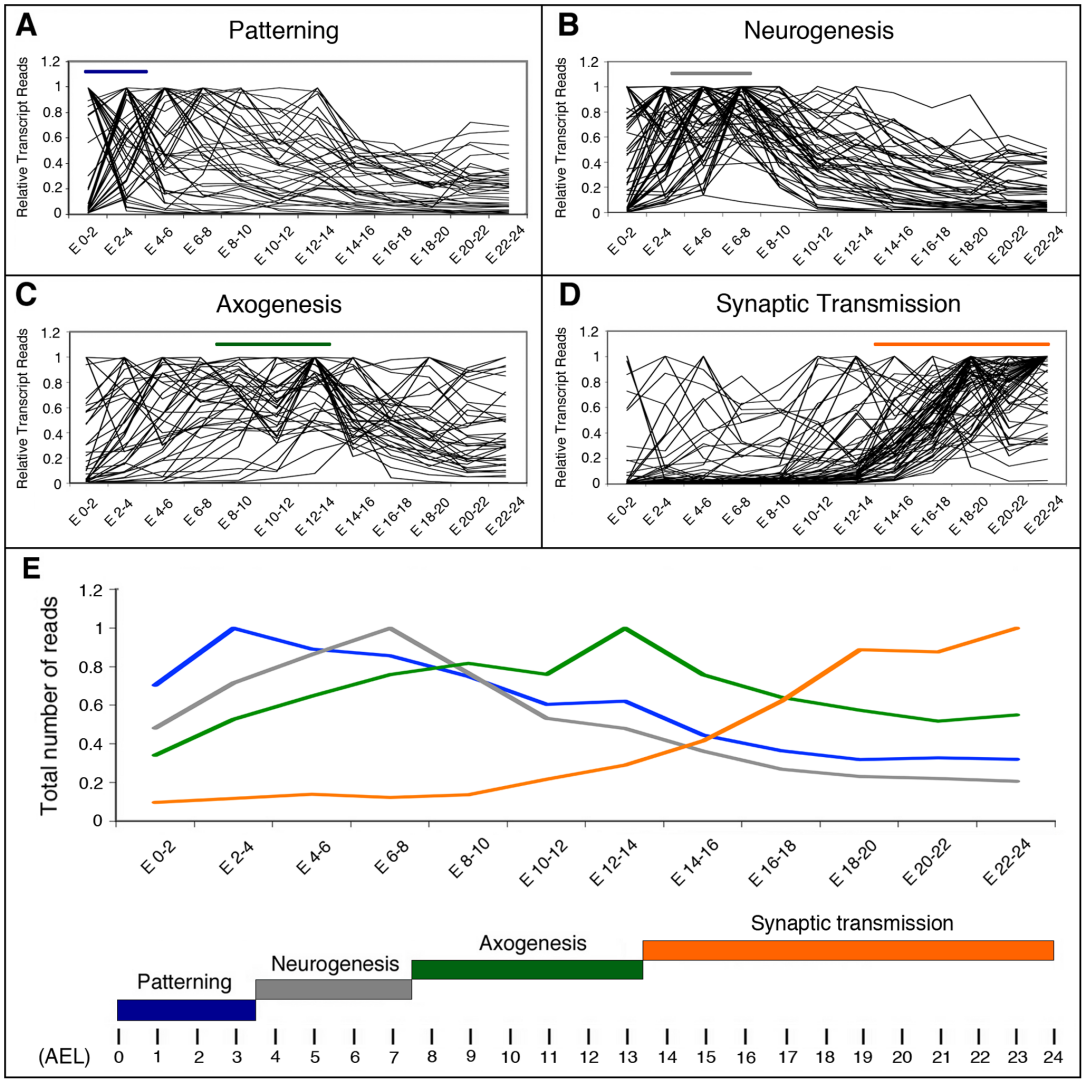
Transcriptional profiles of genes involved in early development and late differentiation of neurons in *Drosophila*. **A–D**: Graphical representation of the expression profiles of individual genes within the indicated sub-catalogues during the specified developmental intervals. Transcriptome data are from Graveley et al. [5]. Horizontal bars show the developmental time when these processes take place following the color code indicated below. **E**: The total number of transcript reads of the genes belonging to the Patterning, Neurogenesis, Axogenesis or Synaptic Transmission sub-catalogues was plotted *versus* the indicated developmental times in a 0 to 1 scale. Bellow, the developmental times when these processes take place are indicated along a time scale of 24 hours. AEL: after egg laying.

The results reported above prompted us to investigate the expression profile of gene catalogues representing particular biological processes of importance for nervous system development and differentiation. Our hypothesis was that a catalogue's profile (i. e. the graphic representation of the catalogue's total transcripts along time) will match the progress of the biological process that it represents. To test this idea we studied the expression profiles of the previously mentioned sub-catalogues, along a temporal series of 12 samples taken at 2-hours intervals during embryogenesis [5]. Our sub-catalogues represent distinct developmental processes known to occur in the embryo along a well-defined temporal sequence: the process of patterning, that defines where the nervous tissue will be formed; the process of neurogenesis, where the neural stem cells are formed and specified; the process of axogenesis, defined by the formation and growth of the axons; and finally, the process of synaptic transmission. Importantly, each of these developmental phases occurs consecutively in time and requires the initiation of the previous one: synapses are formed by axons and dendrites, structures formed exclusively by neurons; neurons are in turn generated by neural stem cells, which develop only in precise regions of the embryo specified by patterning mechanisms. Figure 3 shows the graphic representation of the transcript values for each individual gene within the four sub-catalogues Patterning, Neurogenesis, Axogenesis and Synaptic Transmission (Figure 3A–D), as well as the catalogue's profile (i. e. the total number of transcripts for each catalogue) along time (Figure 3E). Two other sub-catalogues (Synapse Assembly and Ion Channels) showed profiles similar to Synaptic Transmission and for clarity and space are not illustrated here. Our results were coincident with the hypothesis, as each catalogue's profile peaked at the predictable time point in spite of considerable variation in the expression profiles of individual genes.

Among the 46 genes from the Patterning sub-catalogue (Table S1), 13 have a peak of expression in the 0–2 hours AEL interval, 12 in the 2–4 hours AEL interval, 11 in the 4–6 hours interval and the remaining 10 genes in later intervals (Table S2). Therefore, 25 (54%) of the genes included in this sub-catalogue show a peak of expression co-temporal with the time of development when patterning of the embryo is specified (Figure 3A; Table S2). The largest group (13 genes) includes *oskar*, *pumilio*, *nanos*, *bicoid*, *exuperantia*, *caudal*, *torso*, *dorsal*, *tube* and other maternal genes for which high transcript doses are deposited in the egg before fertilization. These genes help to define the major body coordinates during the first hours of embryogenesis [26]. Conversely, most of the genes with later expression peaks (for example *dpp*, *Egfr*, *vnd* or *Dr*) are known to function slightly later for the specification of smaller regions of the neurogenic zone (reviewed by [27]). Some genes (i. e. *capu*) show high transcription levels at early stages and present a second peak of expression 8–10 hours later. When the total reads of the genes from of this sub-catalogue are pooled together, the highest transcript level coincides with the period of embryogenesis when patterning takes place (Figure 3E).

Neurogenesis starts short after gastrulation (3.5 hours AEL), when the action of proneural genes and the Notch pathway promotes the formation of neuroblasts (stem cells), and ends by stage 11 of embryonic development (between 5.20 and 7.20 hours AEL) [28]–[31]. For 45 of the 56 genes from the Neurogenesis sub-catalogue (Table S1A), almost 79%, the peak of expression is coincident with the time of formation and specification of the neuroblasts (Figure 3B; Table S2). Eleven out of these 44 genes show a transcription peak in the 2–4 hours AEL interval, 15 in the 4–6 hours interval and 19 in the 6–8 hours interval (Figure 3B). When the transcript reads of all these genes are pooled, the transcript values showed an increase when neurogenesis begins (approximately in the 2–4 hours AEL interval), a peak in the 6–8 hours interval and then a gradual decrease (Figure 3E), mostly due to genes that have a function in cell specification of neuroblasts and their progeny during this time [27], [31]. The decline in transcripts levels during the last third of embryonic development is coincident with the finalization of neurogenesis and the intensification of differentiation.

Axogenesis in the nerve cord begins around 8 hours AEL [32]–[34] and culminates by 13.5 hours AEL [35]. The temporal expression profile of the Axogenesis sub-catalogue (40 genes, Table S1A) was more complex than that of the sub-catalogues mentioned above (Figure 3C), as it showed transcription peaks in practically every time interval, including 6 peaks in the “pre-axonal” period (i. e. at 0–2 and 2–4 hours AEL, before axons are formed; Figure 3C). In addition, many of the genes from this sub-catalogue showed more than one peak of expression, the first of which occurring in pre-axonal stages and the second when axons are growing. However, in spite of this variability among genes, the pooled transcripts for the entire sub-catalogue coincided in time with the stages of intense axogenesis (Figure 3E; Table S2). Another unexpected feature was that 30% of these genes (i. e. *babo*, *Fas1*, *Fas2*, *lea*, *PlexA*, *PlexB*, *robo*, *Sema-1a*, *stan* and *trio*) showed peaks of expression at 8–10 hours and 12–14 hours AEL intervals, suggesting a possible functional relevance of this expression pattern.

In contrast to this clear temporal sequence in Neurodevelopment subcatalogues of Patterning, Neurogenesis and Axogenesis, in which the profile of each subcatalogue shows temporal correlation with the corresponding biological function, all these subcatalogues share the same profile during postembryonic development. They show a large increase in transcripts during the last larval stage and a single peak at 24 hours APF, similar to the entire Neurodevelopment catalogue (compare Figure 2B with Figure S1).

The last sub-catalogue studied in this section comprised genes necessary for synaptic transmission (Table S1B), a biological function associated with late stages of neuronal differentiation. Among these 72 genes, some were found to have peaks of expression at early stages, before the formation of neurons, but approximately 90% of them had their peak of expression during the second half of embryonic development (Figure 3D), specially in the last third of embryogenesis (82%), when synaptic transmission is initiated (Table S2) [20], [21]. This clear correlation between an increment in gene expression and the developmental window when synapses are formed and start to function is clearly reflected by the profile of the sub-catalogue's pooled data (Figure 3E).

### Correlation of genes from different functional groups

**Figure 4:**
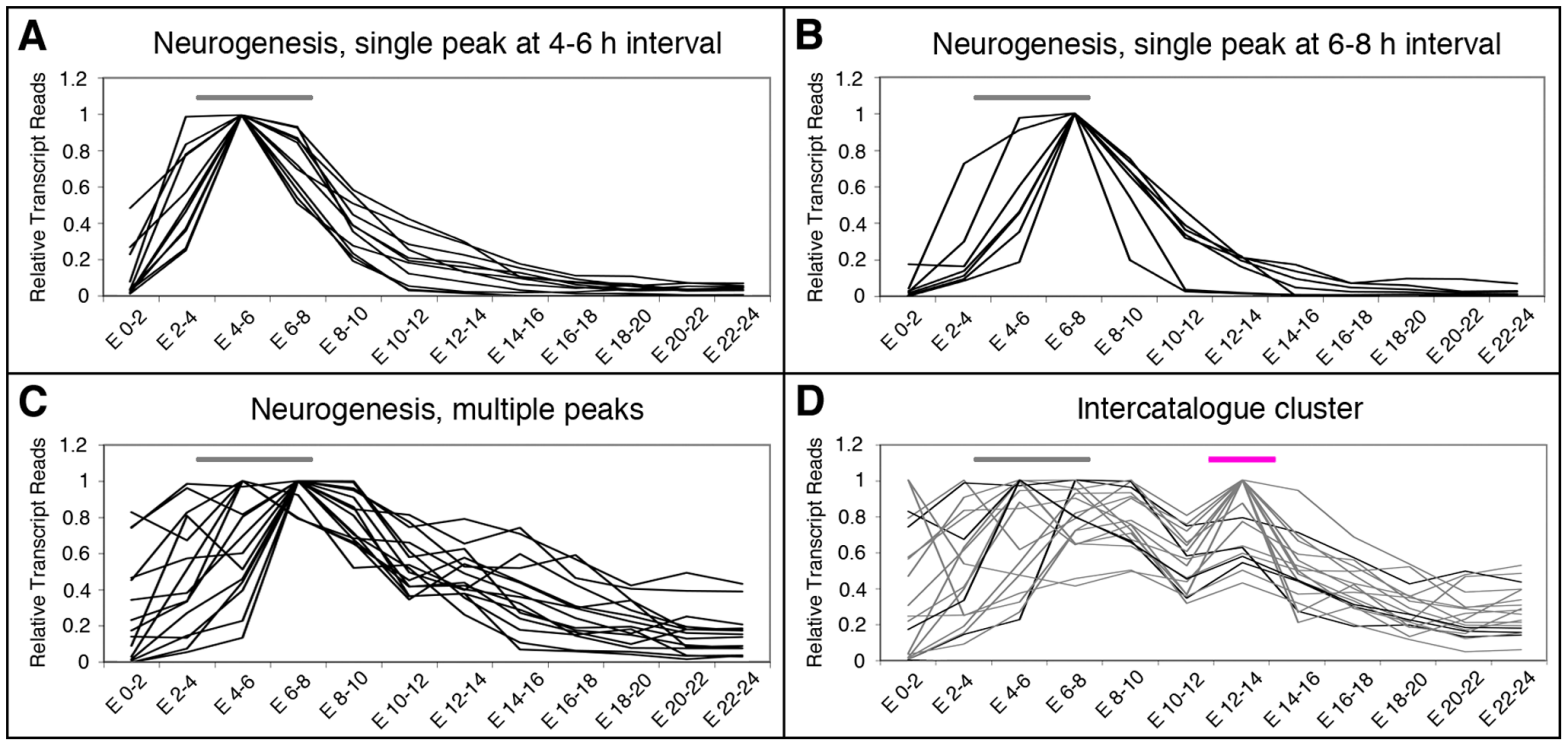
Expression profiles of the genes from the Neurogenesis sub-catalogue. **A–D**: Graphical representation of the three types of expression profiles found for genes from the Neurogenesis sub-catalogue, represented in a 0 to 1 scale. Gene profiles of the type “Single peak of expression at the 4–6 hours AEL interval”, “Single peak of expression at the 6–8 hours AE interval” and “Multiple peaks of expression” are illustrated in A, B, and C, respectively. **D**: Several of the genes with multiple peaks of expression, show a second peak at the 12–14 hours AEL interval, as several others genes from the Patterning and Axogenesis sub-catalogues (Figure 3). The grey horizontal bars in A, B and C indicate the developmental time when Neurogenesis takes place. The pink horizontal bar in E indicates the 12–14 hours AEL interval. Transcriptome data from Graveley et al. [5] were used for the analysis.

Genes with more than one peak of expression during embryonic development have been reported to be relatively frequent [1], [4]. As reported above, many of the genes investigated here had more than one peak of expression along embryogenesis. The second peak could appear at any time interval following the first peak, but most frequently at 12–14 hours AEL. This means, in turn, that at certain time points genes from different catalogues peak together. To illustrate this feature we use the expression profile of the Neurogenesis sub-catalogue. Thirty-four out of the 55 genes in this sub-catalogue have their highest expression at the beginning of neurogenesis, between 4 and 8 hours AEL (15 and 19 genes, respectively; Table S2). Of these genes, 11 have single peaks of expression in the 4–6 hours interval (Figure 4A) and 7 in the 6–8 hours AEL interval (Figure 4B), whereas the remaining genes have additional peaks of expression in other time intervals (examples are shown in Figure 4C). Among the genes with multiple peaks, 4 have a second peak of expression at the 12–14 hours AEL interval and did so together with 12 genes from the Axogenesis sub-catalogue and 3 genes from the Patterning sub-catalogue (Figure 4D). These results suggest that at 12–14 hours AEL of embryonic development, the regulation of genes from different sub-catalogues correlates.

### Experimentally related genes show similar profiles than functional sub-catalogues

**Figure 5:**
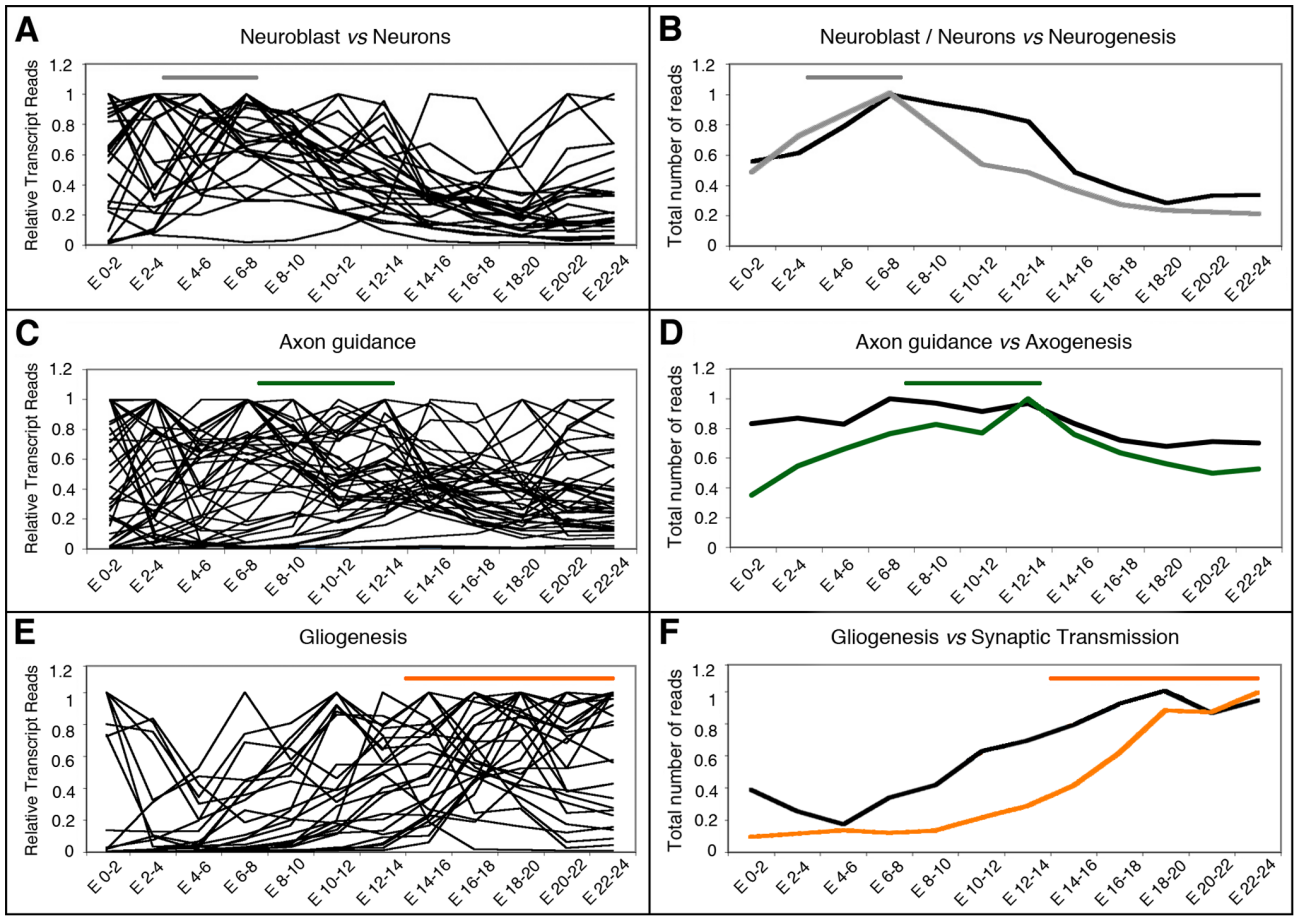
Expression profiles of functionally related genes compared with the Neurogenesis, Axogenesis and Synaptic Transmission sub-catalogues. **A, C, E**: Graphical representation of the expression profiles of individual genes from functional catalogues defined experimentally in studies designed to identify genes with differential expression profiles in neurons *vs* neuroblasts [36] (**A, B**), axonal guidance [37] (**C, D**) and glial cells [38] (**E, F**). **B, D, F**: The total number of transcript reads of the genes selected experimentally (black lines) were plotted versus the specified developmental intervals represented in a 0 to 1 scale and compared with the total number of transcript reads of genes from the Neurogenesis, Axogenesis or Synaptic Transmission sub-catalogues. Lines have the same color code as in Figure 3. Horizontal bars indicate the developmental time when these processes take place following the color code as in Figure 3. Transcriptome data from Graveley et al. [5] were used for the comparison.

Next, we tested whether our approach can be applied to catalogues of genes defined by genomic studies designed to identify genes associated with specific biological processes. In a study comparing the transcriptome of proliferative (stem cells) *versus* post-mitotic, differentiated cells (neurons) in the nervous system of the *Drosophila* larva, Berger and collaborators defined a set of 28 genes that encode transcription factors which are spatially regulated within the nervous tissue: they are upregulated in stem cells but downregulated in differentiated neurons (Figure 4A in [36]). If these genes have the same function in the embryo and their transcripts are temporally co-regulated, they should be upregulated at the time of neuroblast proliferation and downregulated during neuronal differentiation. To test this hypothesis we examined the number of reads of these 28 genes throughout embryonic development in the temporal data series published by Graveley et al. [5]. The majority of these genes (19 out of 28) showed lower expression during the last third of embryonic development, when neurogenesis is downregulated and neuronal differentiation predominates (Figure 5A). When the total number of transcript reads of all these genes was plotted, it showed a clear correlation between high levels of expression during proliferation and low levels of expression during differentiation, and closely resembles the profile of our Neurogenesis sub-catalogue (indicated by a grey line in Figure 5B).

We also tested a catalogue of 47 genes obtained from a screening designed to detect genes important for axonal guidance (Table 2 in [37]), comparing their expression profile with that of our Axogenesis sub-catalogue. Several features were strikingly similar between the profiles of both catalogues: a large proportion of the 47 genes had more than one peak of expression; groups of genes peaked together in 9 out of the 12 temporal intervals, including several peaks during pre-axonal stages; about half of these genes contributed to the major peak of expression in the 12–14 hours AEL interval; and also showed a decline in transcripts in the 10–12 hours AEL interval (Figure 5C). This last feature left a clear trace in the profile of the pooled transcript reads from all the genes of the axonal screening, which was similar to that of our Axogenesis sub-catalogue (indicated with a green line in Figure 5D).

Lastly, we tested a catalogue of 26 genes that are expressed exclusively in glial cells during embryonic development (Table 2 and 3 in [38] and personal communication). We found that the expression profiles of these genes most closely resembles those of our Synaptic Transmission sub-catalogue (Figure 5E, F), especially during the second half of the embryonic life as it is shown in Figure 5F where profile of the total transcripts of the Gliogenesis catalogue (black line) coincides with that of our Synaptic Transmission sub-catalogue (orange line).

### Gene Ontology term enrichment in correlated expression profiles

**Figure 6:**
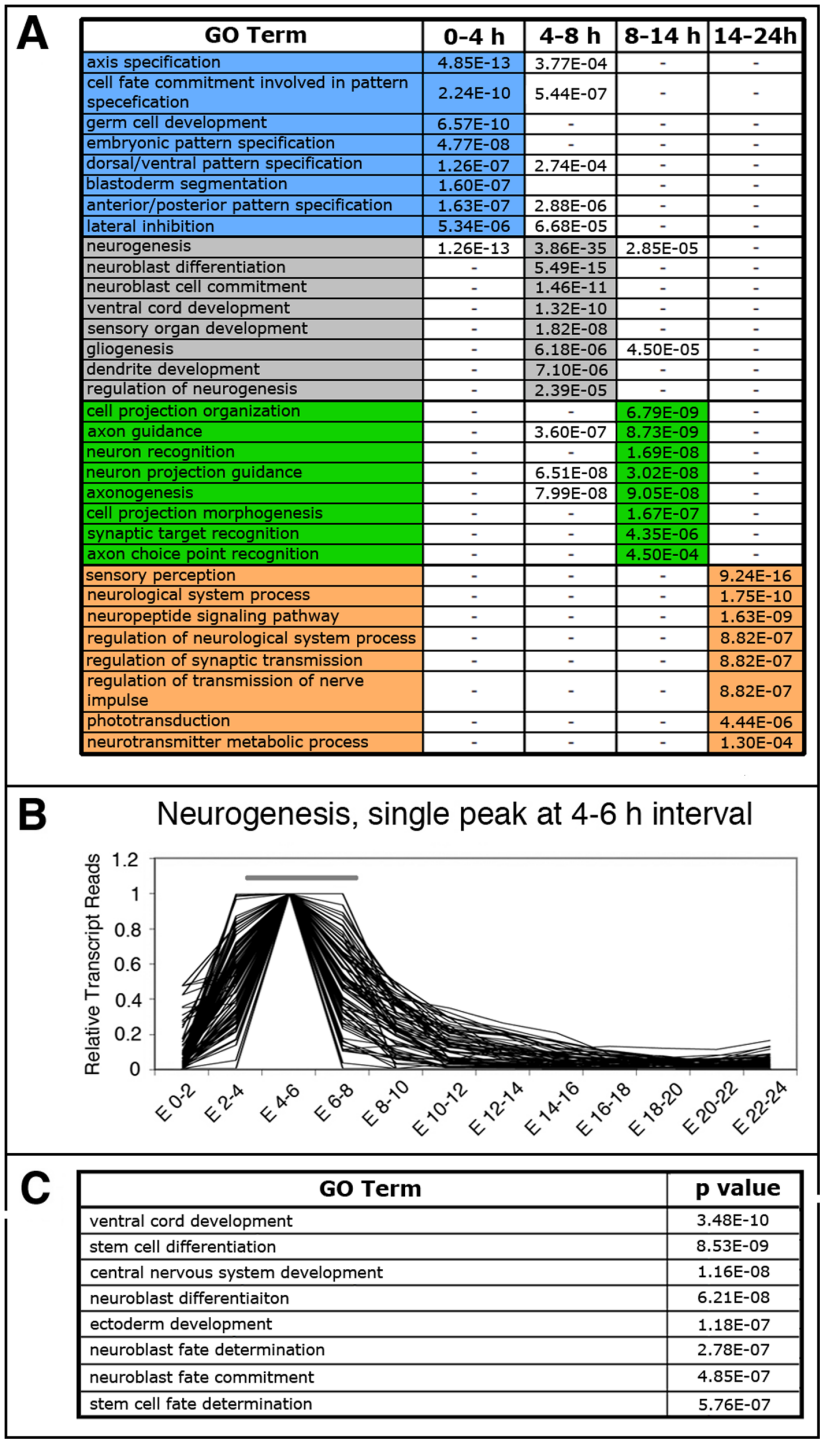
GO term enrichment analysis and functional prediction. **A**: GO terms enriched in the specified developmental intervals. Color code is as in Figure 3. P values are indicated, the lowest one per GO term are colour-highlighted. (-) indicates that no significant enrichment was found. **B**: Graphical representation of 81 genes selected from the whole *Drosophila* genome using data from Graveley et al. [5] based on their transcriptional profiles, having a single peak in the 4–6 hours AEL interval in a 0 to 1 scale. **C**: GO terms enriched the list of genes plotted in B. P values are indicated.

Our previous results indicate that functionally related genes have frequently similar expression profiles during embryonic development. If so, groups of genes with similar expression profile might be enriched in functional Gene Ontology (GO) terms associated with the specific developmental stage where the expression peaks occurs. To test the predictive capacity of our hypothesis, we used VLAD to analyze the lists of genes in the *Drosophila melanogaster* genome that show highest expression levels in the following intervals according to Graveley et al. data (Table S9; [5]): the 0–4 h AEL interval that includes most of the genes from our Patterning sub-catalogue, 3984 genes in the entire *Drosophila* genome; the 4–8 h AEL interval that includes most of the genes from our Neurogenesis sub-catalogue, 1872 genes in the *Drosophila* genome; the 8–14 h AEL interval that includes most of the genes from our Axogenesis sub-catalogue, 2194 genes in the genome; and the 14–24 h AEL interval that includes most of the genes from our Neurodifferentiation catalogue, 5346 genes in the genome. According to our hypothesis, these new predictive catalogues should be enriched in genes involved in terms related to the biological function assigned for the particular time interval of the embryonic development considered. In fact, the list of genes showing the highest peak of expression in each one of these intervals was enriched in the expected GO terms (Figure 6A and Table S3). Thus, the GO term “axis specification” is predominantly enriched in the 0–4 h AEL interval; the term “neurogenesis” is predominantly enriched in the 4–8 h AEL interval; the term “axon guidance” is predominantly enriched in the 8–14 h AEL interval; and the term “synapse” appears in the 14–24 h AEL interval. These results correlate well with those obtained with our functionally biased catalogues.

We further rationalized that smaller lists of genes with a more defined expression profiles should be enriched in particular functional GO terms. To exemplify this, we chose one of the expression profiles shown in Figure 4A, which represents those genes that have a single peak of expression in the 4–6 h AEL interval. We searched in the *Drosophila melanogaster* genome for genes that show the same expression profile (a peak in the 4–6 h AEL interval) and selected a list of 81 genes (Figure 6B and data not shown). GO analysis of this list show an enrichment in terms related to neuroblast specification and neuronal formation (Figure 6C), which correlates well with the neurogenic function that takes place in the embryo at this time interval. Of those 81 genes, 8 genes were included in our Neurogenesis sub-catalogue. The involvement in neurogenesis of the remaining 73 genes should be checked experimentally.

## Discussion

Developmental changes are associated to changes in the transcriptome. Making use of the great amount of data derived from genomic studies, the transcriptional profile of groups of genes involved in the same developmental processes can be analyzed. We showed here that during *Drosophila* embryonic development the expression profile of functional catalogues correlates with the temporal windows of the biological processes they represent and that many of the genes within each catalogue have similar expression profiles during development.

By predefining relatively small but functionally homogeneous gene catalogues, we could observe coherent changes in gene expression out of genomic data obtained from whole embryos even for gene catalogues representing biological processes highly restricted in time and space, as the formation of neural stem cells in the neuroectoderm or the formation of neuronal branches and synapses in the nervous tissue.

The relevance of this approach is reflected by the fact that the transcriptional shift that occurs in the embryo, from a predominant expression of genes mainly associated to the early neurodevelopmental processes to one of those genes mainly associated to late neurodifferentiation, as well as the correlated sequence of temporal expression profile of gene catalogues representing even smaller developmental processes had not been previously observed. It appears that algorithms used in previous studies to detect coherent changes in gene expression along time increase the likelihood of defining relatively large groups (often hundreds of genes) with relatively poor functional resolution [1]–[4].

Once we obtained evidence for the potential of this approach, we used it to investigate other developmental stages and discovered a second transcriptional shift from early neurodevelopment to late neurodifferentiation between the first and third day of pupal life. From later larval to late pupal stages the nervous system undergoes a dramatic reorganization [39]. However, neither the profile of the Neurodevelopment catalogue nor that of its subcatalogues showed a temporal sequence in which each catalogue's profile had clear temporal correlation with the corresponding biological process. Postembryonic neurogenesis starts at the end of the first day of larval life and continues to the beginning of pupal life [24], [40], [41]. The neurons generated during this time remain in a rather undifferentiated state until the beginning of metamorphosis, when they all enter axogenesis simultaneously [25]. In spite of this sequence of biological events, the subcatalogues for Neurogenesis and Axogenesis show the same expression profile during this phase (Figure S1). This profile is also shared by the remaining subcatalogues (Dendrite morphogenesis and Miscellaneous; Figure S1), suggesting that during postembryonic development these genes are co-regulated in a qualitatively different way than during embryonic development. The different patterns in transcriptional regulation might be related to the differences in temporal scale. In the embryo, development is so quick that changes in transcription most probably reflect direct changes in protein levels. In postembryonic development, when each phase might prolong for several days, additional regulatory mechanisms might blunt the sharp correlations between gene transcription and biological function observed in the embryo.

Among the problems intrinsic to the analysis of *Drosophila* genomic data generated from whole-organisms is that the resolution might be dulled by the existence of genes with more than one peak of expression during development [1], [4], the existence of pleiotropic genes that may be up- or downregulated at different times in different tissues, and the possibility that small but biologically relevant changes in expression cannot be detected because they are filtered out by the statistical frame used for the bioinformatics analysis.

In the profile of total transcript reads of genes from the Axogenesis sub-catalogue we found an “indentation” or ground “trough” at 10–12 hours AEL (Figure 3E). This feature was also observed in a catalogue of genes of relevance for axonal development defined by others (Figure 5D; [37]). Nevertheless, we believe that this feature, rather than being exclusive for the axogenic process, reflects a global transition in the embryo's transcriptome. As shown in Figure 4D, we also found this feature in the expression profile of some genes from other sub-catalogues. Interestingly, a massive shutdown/restart of gene expression around this time was reported by others [2], [3]. Such a global regulation of gene expression might be controlled by hormonal signals. It appears worth noticing here that a few hours before there is a surge in ecdysone [42], a steroid hormone known to coordinate many aspects of gene expression, morphogenesis and substantial changes in physiology and behavior along the life cycle of *Drosophila* [43], [44].

As shown here for the catalogue of 28 genes with higher expression in proliferative than in differentiated neurons during *Drosophila* larval life [36], the expression profiles of these genes in the embryo correlated with the timing of two broad functions (proliferation *versus* differentiation), even when the functional catalogue was defined with RNA samples collected from a different developmental stage. This reinforces the view that a functionally biased approach can enrich the study of coherent waves of genes expression along an organism life cycle.

The resemblance in the expression profiles of gene catalogues specific for either glia cells or synaptic transmission is remarkable and here we suggest two explanations. The simplest one will be that it reflects just temporal correlation between differentiation of synapses and glial cells without mutual functional relevance. A second explanation will take into account what is known about the functional relationship between glia and neurons. Glia cells contribute in numerous ways to neuronal development participating in an array of functions at different times, from neuroblast proliferation early on, to axonal growth in mid-stages and very late in development when they insulate axons and make the blood-brain barrier necessary for the propagation of the nerve impulse towards synapses (reviewed in [45], [46]). Yet, we found that the expression profile of the glia-specific catalogue most closely resembles that of our Synaptic Transmission sub-catalogue. The importance of glial cells for synapse formation is well established [47] but, whereas some of the genes of the Synaptic Transmission sub-catalogue are expressed in neurons and other cells, those in the glia catalogue are expressed exclusively in glial cells [38], suggesting that in spite of their different spatial regulation, both sets of genes share temporal co-regulation during the last phase of embryogenesis.

The predictive capacity of the method is reinforced by the analysis shown in Figure 6. A list of genes with common expression profile is enriched in GO terms corresponding to biological processes developed during that particular time interval (Figure 6B, C). Some of the genes from these lists have not reported function or are assigned to other GO terms not related to neurogenesis. We observed that 77% of these genes are expressed in neurogenic tissues and that a neurogenic function has been either reported or indicated for 16% of them in the scientific literature (Table S4), indicating that this gene catalogue contains neurogenic genes for which that function is yet unexplored.

We believe that the examples documented in this study show that our approach could be applied to other processes of developmental biology, at least if working with embryonic samples and temporal data series of the quality tested here. Recent advances in deep sequencing technology [48]–[50] will probably lead to a rapid increment in the quantity and quality of the data collections available for this type of studies. Perhaps a major goal will be to use the particular features in the expression profile of a functional catalogue as an aid to search for genes yet not annotated for that function. This will facilitate the selection of genes to be experimentally investigated with regards to specific biological processes important for neural development, as well as for other important processes.

## Materials and Methods

### RNA sequencing data

The data used in this study were previously published [5], [23]. Ferreiro et al. compared the deep-sequenced transcriptome of a *Drosophila melanogaster* wild type strain (Vallecas) at stages 16 and 17 of embryonic development. Graveley et al. deep-sequenced the transcriptome of *Drosophila melanogaster* isogenic (*y^1^*; *cn bw^1^ sp^1^*) embryos collected at 2-h intervals for 24 h. The 0 to 1 scale data were generated by considering the highest value among the number of reads for each transcript as 1 in the embryonic temporal series. The rest of the values for that transcript was calculated accordingly.

### Generation of the catalogues

We used FlyBase [51] and the scientific literature to assemble catalogues of *Drosophila* genes which contribution to a particular biological function was documented by means of mutant phenotypes or other experimental approaches (Table S1 shows the main references for each gene). Complete gene coverage of the catalogues was not attempted since preliminary tests showed that even smaller catalogues gave similar results (Figure 1B and data not shown). Which and how many genes were included in each catalogue was determined ad hoc. For the catalogue Neurodevelopment (biased towards early development) we included 200 genes relevant for different developmental aspects: proneural genes; genes from the Notch and Dpp pathways; genes important for the specification of stem cells; axon growth; axon guidance and dendrite morphogenesis; and a miscellaneous group, including for instance genes of relevance for glial *versus* neuronal cell fate (Table S1A). For the catalogue of Neurodifferentiation we selected 200 genes biased towards the latest steps of embryonic development, such as: synapse assembly genes; synaptic vesicle dynamics genes; ion channels genes; neurotransmitters genes and those encoding for their receptors (Table S1B). Both catalogues include genes for which other functions are known, not related with the hypothesis under study. For instance, the Neurodevelopment catalogue includes the pleiotropic genes *dpp*, *engrailed* and *Notch*, which are also expressed by non–neural tissues. The Neurodifferentiation catalogue includes genes corresponding to ion channels also expressed by non-neuronal tissues and the gene encoding the enzyme *dopa decarboxylase*, which is also expressed by the epidermis as a part of the biochemical pathway of cuticle secretion.

The sub-catalogues for Patterning, Neurogenesis and Axogenesis were done in the same way and are presented in the Table S1. As explained above, many of the genes in these sub-catalogues might have several functions, often in tissues different than the nervous system. GO analysis was done using Gather [52] to further confirm that each catalogue was clearly enriched in the biological annotations corresponding to the functions it represents.

The catalogue of 28 genes that encode transcription factors upregulated in neural stem cells and downregulated in neurons during *Drosophila* larval life and the catalogue of 44 genes proposed to be important for axon development, were taken from their original publications [36], [37]. The catalogue of 26 genes expressed exclusively in glial cells during *Drosophila* embryonic development was provided by Dr. Altenhein [38].

For the GO analysis of the genes that peak at different developmental intervals we used, VLAD (Visual Annotation Display, http://informatics.jax.org/~jer/vlad-1.0.3). Similar results were obtained using Gather [52] or FlyMine (http://www.flymine.org) online databases.

### Graphical representation of expression profiles

For the catalogues of Neurodevelopment and Neurodifferentiation we scored the transcript values for every gene and compared them at stages 16 and 17 of embryonic development using the two RNA sequencing data sets mentioned above [5], [23]. For the remaining catalogues we plotted the transcription level values (number of sequencing reads) for each gene [5] along the developmental times, to produce a graphic representation of the putative expression profile.

## Acknowledgments

We thank Joachim Urban, Christian Berger and Benjamin Altenheim for the critical reading of the manuscript. We also thank insights and suggestions provided by the two referees and the Academic Editor, which substantially improved the manuscript.

## Supporting Information

Figure S1 **Transcriptional profiles of genes involved in** ***Drosophila*** **neural development during late larval and pupal stages.** Graphical representation of the pooled expression profile of each of the five subcatalogues of Neurodevelopment used in this study (See Table S1A). During postembryonic life, instead of a temporal sequence in which the profile of each subcatalogue reflects the timing of the corresponding biological process (compare with Figure 3E), all these sub-catalogues share the same profile, with a large increase during late larval and early pupal life, with a peak at 24 hours APF.
(TIF)

Table S1 **Gene Catalogues. A**: Neurodevelopment. **B**: Neurodifferentiation.
(XLS)

Table S2 **Peak of expression for every gene in each catalogue in a 0 to 1 scale.**
(XLS)

Table S3 **Genome-wide catalogues of genes with peaks of expression at different developmental intervals.** Each page shows the genes with the highest peak of expression at the indicated time intervals: Patterning, 0–4 h AEL; Neurogenesis, 4–8 h AEL; Axogenesis, 8–14 h; Neurodifferentiation, 14–24 h. The genes marked in bold are those that were members of our sub-catalogues for Patterning, Neurogenesis, Axogenesis or Neurodifferentiation (Table S2).
(XLS)

Table S4 **Neurogenesis prediction analysis.** Expression data for the 73 genes found in the Neurogenesis prediction analysis (sheet Neurogenesis in Table S3), extracted from modENCODE, FlyAtlas and BDGP. When available, CNS phenotypes reported by FlyBase and references to publications reporting or suggesting involvement of the gene in neurogenesis are also shown.
(XLSX)
