# Supplementary figures and images for: Global Gene Expression Shift during the Transition from Early Neural Development to Late Neuronal Differentiation in Drosophila melanogaster
Source: PLoS One. 2014 May 15;9(5):e97703. doi: 10.1371/journal.pone.0097703 (PMC4022633; doi:10.1371/journal.pone.0097703)

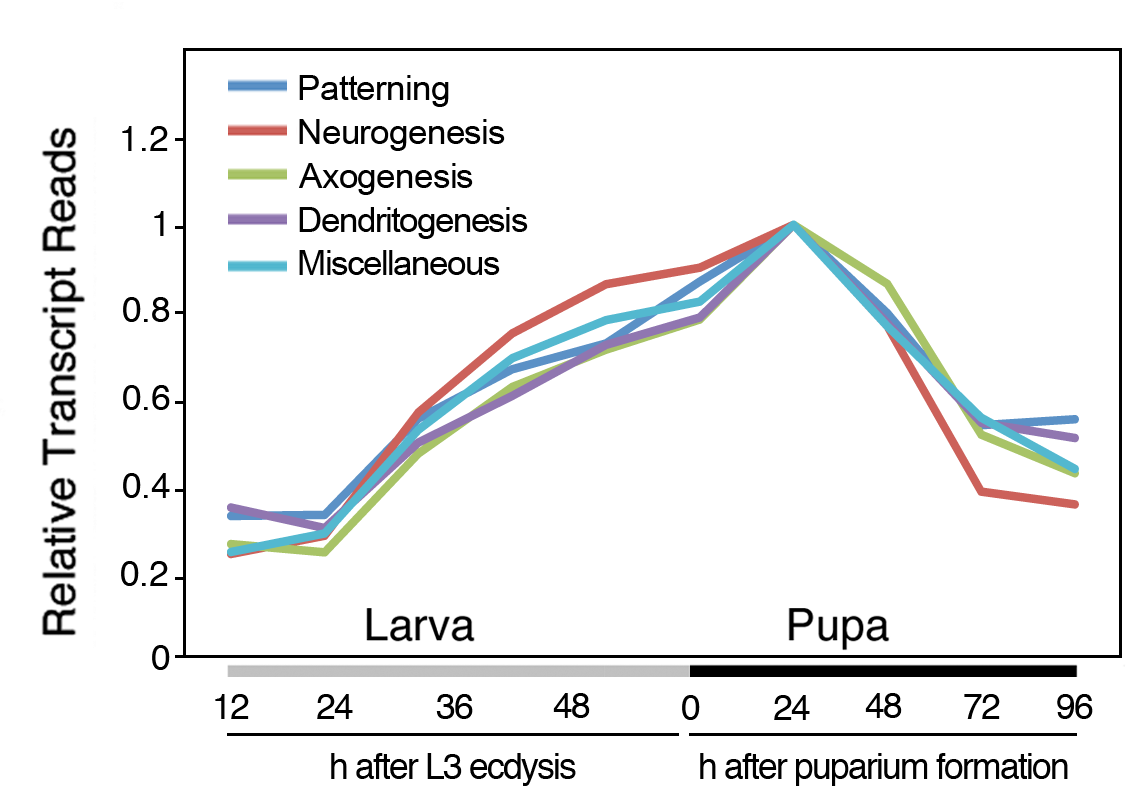

Supplement: Figure S1 — Transcriptional profiles of genes involved in Drosophila neural development during late larval and pupal stages. Graphical representation of the pooled expression profile of each of the five subcatalogues of Neurodevelopment used in this study (See Table S1A). During postembryonic life, instead of a temporal sequence in which the profile of each subcatalogue reflects the timing of the corresponding biological process (compare with Figure 3E), all these sub-catalogues share the same profile, with a large increase during late larval and early pupal life, with a peak at 24 hours APF. (TIF) [file pone.0097703.s001.tif]
